# Attitudes of Healthcare Service Users in Bulgaria Towards the Application of Teleophthalmology in the Case of Glaucoma

**DOI:** 10.3390/healthcare14020273

**Published:** 2026-01-21

**Authors:** Stanka Uzunova, Rumyana Stoyanova, Marin Atanassov, Kristina Kilova

**Affiliations:** 1 Department of Health Management and Health Economics, Faculty of Public Health, Medical University of Plovdiv, 4002 Plovdiv, Bulgaria; suzunova@doctor.com; 2 Department of Ophthalmology, Faculty of Medicine, Medical University of Plovdiv, 4002 Plovdiv, Bulgaria; marin.atanassov@mu-plovdiv.bg; 3 Department of Medical Informatics, Biostatistics and E-learning, Faculty of Public Health, Medical University of Plovdiv, 4002 Plovdiv, Bulgaria; kristina.kilova@mu-plovdiv.bg

**Keywords:** glaucoma, teleophthalmology, teleglaucoma, users, healthcare services

## Abstract

**Objectives**: The purpose of the current research is to examine and analyze the attitudes of healthcare service users towards the integration of remote medical services into ophthalmology in Bulgaria, including teleglaucoma. **Methods**: A cross-sectional survey study was conducted among 902 healthcare users during the period from May 2023 until December 2024. Descriptive statistics, parametric, and non-parametric tests for hypothesis testing were used. **Results**: The present study outlined predominantly positive attitudes towards the use of telemedicine services in ophthalmology, with 69.6% of respondents reporting a positive overall opinion in the final assessment. The greatest support was observed during remote consultations with a familiar doctor (77.4%) and during continuous follow-up of eye conditions (55.2%). Willingness to use such services was lower in emergencies or when contacting an unfamiliar specialist. A significant correlation was established between socio-demographic characteristics and attitudes—respondents with greater education levels (*p* = 0.006), men, and younger participants were more positive towards telemedicine (*p* < 0.05). The high level of awareness about glaucoma, particularly among those with university-level education, served as a positive prerequisite for the implementation of teleophthalmology services related to its monitoring. Mobile applications and digital solutions were evaluated as beneficial means of facilitating communication and increasing adherence to treatment. Regarding the use of artificial intelligence, certain skepticism and insufficient awareness levels were observed, which required additional efforts to increase trust and digital literacy among users. **Conclusions**: The implementation of telemedicine services into ophthalmology has potential but outlines the necessity of considering the individual attitudes of applying coherent quality and safety standards and of directed awareness campaigns, especially towards the groups of lower technological and healthcare literacy.

## 1. Introduction

Managing chronic eye conditions is a challenge that healthcare systems face. The population is increasing and aging, which increases the necessity of eye care. Data showed that in 20 years of non-interference, the number of visually impaired people globally has increased from 44 million in 2000 to 76 million in 2020. In 2020, in Bulgaria, there were 1.3 million people who had lost their sight, and 20,000 of them were blind [1]. Internet-based eye care-teleophthalmology has been considered a solution to this issue, via which eye care could be at the disposal of everyone in need [2].

Teleophthalmology is a branch of telemedicine that renders eye care via digital medical equipment and telecommunication technologies. Today, the teleophthalmology applications include access to eye specialists for patients in remote areas, screening for ophthalmologic conditions, diagnostics, treatment, follow-up, and observation, as well as remote training [3]. The specialists have the opportunity to provide consultations in flexible schedules and locations, including at their homes. Teleophthalmology offers short examination times, digital medical images, and the opportunity for other specialists to perform screening for conditions, as well as secondary advice from specialists during diagnostics and management of difficult cases [2]. In clinical practice, teleophthalmology services are delivered through various operational models, including synchronous (real-time), asynchronous (store-and-forward), and hybrid approaches. Asynchronous models are widely used for screening and long-term follow-up, as they allow remote evaluation of images and clinical data collected by trained personnel. Synchronous models involve direct communication between the patient and the specialist through video consultations. Contemporary international programs demonstrate that these models improve access to ophthalmic care and provide clinical effectiveness comparable to that of traditional in-person examinations [4].

Teleglaucoma is an integral part of teleophthalmology and perhaps one of the most challenging areas since the glaucoma disease requires various functional tests, continuous observation, and analysis of the results. It has great potential to improve the patient’s condition and quality of life via access to high-quality and cost-effective glaucoma care.

Early research showed that telemedicine technologies and portable devices for image diagnostics could support the early discovery, screening, and management of glaucoma, especially in patients with limited access to ophthalmological help [5,6,7]. These results showed that teleglaucoma had the potential to improve clinical results and the patients’ quality of life via timely and economically effective care.

Following the COVID-19 pandemic, teleophthalmology has acquired an increasing impact as a routine component of healthcare. Contemporary research showed that teleophthalmological services were related to high satisfaction of patients, improved access to medical care, and maintained clinical effectiveness in various eye diseases. According to research from East Taiwan, favorable health results and continuous ophthalmological care via teleophthalmology were recorded [8]. Systematic reviews additionally confirmed the effectiveness of teleophthalmology in reaching vulnerable and underserved populations by underlining its role in overcoming geographical and organizational barriers [9].

Awareness and attitudes of the health service users in telemedicine vary considerably depending on age, educational level, and digital literacy. Studies conducted in Italy established that despite the rising awareness, considerable concerns existed, related to the quality of medical care, protection of personal data, and the character of communication between doctor and patient [10]. These results underlined the necessity of preliminary research on the attitudes of patients before widespread incorporation of teleophthalmological services.

Despite the increasing volume of international scientific data, research focusing on patient attitudes towards teleophthalmology in Bulgaria remains limited. This is to a great extent due to the fact that telemedical and particularly teleophthalmological services in the country are not yet widespread and are not systematically integrated into routine clinical practice. There is a lack of national programs, established models of implementation, or clearly defined standards for remote ophthalmological care, including for chronic conditions such as glaucoma. In this context, there are also no systematic analyses to evaluate the readiness of health service users to use such solutions, as well as their perceptions of digital instruments, mobile health applications, and artificial intelligence.

The aim of the present study is to examine and analyze the attitudes among healthcare service users towards the implementation of remote medical services into ophthalmology in Bulgaria, including teleglaucoma.

## 2. Materials and Methods

The current research was an observational, cross-sectional survey study among 902 health service users, conducted in the period May 2023–December 2024. The research had an exploratory character and was directed at an evaluation of the attitudes towards telemedicine in ophthalmology, focusing on teleophthalmology and teleglaucoma, in the conditions of a limited integration of such services in the country.

The inclusion criteria entailed individuals who had reached the age of 18 who self-declared the presence of at least one of the following risk factors: diagnosed with glaucoma, established higher intraocular pressure without therapy, or positive family history for glaucoma. Excluded were individuals under the age of 18, those with cognitive dysfunctions that would interfere with completing the questionnaire, as well as those who did not satisfy any of the listed criteria. Due to the survey nature of the study and the focus on patients’ attitudes and perceptions, affiliation to risk groups was determined based on self-reported information, without clinical verification.

The survey study was conducted online on the Google Forms platform. The link to the questionnaire was distributed via direct sharing with patients at the ophthalmology practice, via electronic messages and social networks, as well as by using the snowball method, where the initial participants were encouraged to share the survey with their friends and relatives, especially those with a family history of glaucoma. Choosing this method was determined by the lack of national registries and the limited development and implementation of teleophthalmology services in Bulgaria, which made it difficult to identify the target population in any other way. Due to the exploratory nature of the study and the absence of previous national data on the topic, the sample size was determined on the principle of including the maximum possible number of participants within the specified study period. The total number of 902 respondents was considered sufficient to conduct statistical analyses and identify major trends in the attitudes of the studied population.

For the purposes of the study, an original survey questionnaire was developed, containing 29 questions. Before answering the questions, participants were provided with a brief standardized description of the concept of teleophthalmology, formulated in clear and neutral language to ensure a consistent understanding of the studied technology across all respondents. The first part included socio-demographic characteristics, and the second part comprises questions related to attitudes towards telemedicine and teleophthalmology, willingness to use remote ophthalmology services, perceptions of mobile health applications, and attitudes towards the use of artificial intelligence in ophthalmology. The introductory part included a design of the purpose of collecting perceptions, namely that the results were necessary solely in relation to studying the attitudes towards the use of medical services remotely. Also specified were instructions for completing the survey, emphasizing that the survey is anonymous, completely confidential, and no personal information would be collected. The data would be used solely for scientific purposes and processed in compliance with the principles of confidentiality and protection under current legislation. Those questions that related to the degree of agreement of the participants were formulated in the form of statements, assessed using a five-point Likert scale, with the possible answers being ‘yes’, ‘somewhat yes’, ‘cannot decide’, ‘somewhat no’, and ‘no’. The questionnaire was prepared based on a review of the existing scientific literature and similar international studies. Before the main study, pilot testing was conducted to assess the clarity, comprehensibility, and logical consistency of the questions. Based on the feedback obtained during the pilot phase, minor revisions were made to the wording and structure of the questionnaire.

The full questionnaire is provided in Supplementary File S1.

The study complied with established standards and adhered to the requirements of the Declaration of Helsinki on ethical principles for medical research and the principles of Good Clinical Practice. It had been approved by the Research Ethics Committee at the Medical University of Plovdiv (Opinion Р-КНЕ-13/14.04.2025).

### Statistical Analysis

The data were analyzed using descriptive statistics, along with parametric and non-parametric tests to verify hypotheses. Central tendency measures were reported as mean (M) and standard deviation (SD). Qualitative data were presented as absolute frequencies and relative percentages. Associations between categorical variables were examined using Pearson’s chi-square test (χ^2^) for multi-dimensional tables and Fisher’s Exact Test for 2 × 2 tables. Analysis of variance (one-way ANOVA) was applied to compare normally distributed quantitative indicators across more than two groups. A *p*-value of less than 0.05 was considered statistically significant for rejecting the null hypothesis.

The data were statistically processed using the software products SPSS 23 and MS Excel 2016.

## 3. Results

A total of 746 women (82.7%) and 156 men (17.3%) participated in the study. The mean age was 40.69 ± 12.55 years, aged 18–77 years. Most respondents (81.8%) were of working age (25–64 years). A large majority lived in urban areas (90.6%; n = 817), and most resided in Bulgaria (96.1%; n = 867).

According to the educational background of the surveyed healthcare service users, one-quarter (n = 225; 24.9%) had completed secondary education, while three-quarters held a higher education degree. Nearly half (42.8%) had a master’s degree, and 6.2% held a doctoral degree.

Analysis of responses from healthcare service users revealed that they were generally positively inclined toward the use of telemedicine services. Table 1 presents responses to key questions concerning users’ attitudes toward these services.

Positive attitude towards the use of telemedicine services in ophthalmology was expressed by 51.5% of the participants (n = 464); this group included those responding ‘yes’ and ‘somewhat yes’. Negative attitude (‘no’ and ‘somewhat no’) was expressed by 26.2% (n = 236), and 22.4% (n = 202) remained hesitant. A statistically significant correlation was established between gender and attitudes towards telemedicine (*p* < 0.001), as men showed a higher willingness to use remote services in comparison to women (65.4% against 48.5%) (see Table 2). Educational level also showed considerable influence (*p* < 0.001) as the highest part of positive attitudes was observed among the respondents with a doctoral degree (64.3%, n = 30) (see Table 3). The age analysis showed a statistically significant correlation (*p* < 0.05), as older participants more often expressed hesitation, depending on the specific telemedicine scenario (see Table 4).

In response to a question about consultations for eye-related issues, 62.5% of respondents (n = 564) answered affirmatively: 100 more than those willing to use telemedicine in general. The analysis showed a significant relationship between age and attitude, with younger participants showing more favorable views (*p* = 0.001) (see Table 4). Gender was also significantly associated (*p* = 0.019): 73% of men answered positively versus 60.4% of women (see Table 2). Education significantly impacted attitudes (*p* = 0.006), with the highest positivity among respondents with a master’s (68.4%) or doctoral degree (60.7%) (see Table 3).

When asked about the possibility of prescribing and initiating treatment for an eye condition through telemedicine, 48.4% (n = 436) responded affirmatively. A statistically significant difference was found with respect to place of residence (*p* < 0.001): urban residents were more likely to favor remote treatment (50.6%), whereas rural residents more frequently expressed disapproval (54.7%). Educational attainment was also a significant factor (*p* = 0.001): respondents with a master’s degree demonstrated the highest level of acceptance (54.5%) (see Table 3).

In cases where there was no prior acquaintance with the consulting physician, 52.6% of respondents reported they would not use a telemedicine service—88 more than those who gave a negative response to the general telemedicine question. Additionally, 16.6% expressed uncertainty, indicating a potential need to raise awareness. A statistically significant association was found regarding gender (*p* < 0.001): men were more willing to accept such a consultation (46.2%) compared to women (27.7%) (see Table 2).

The trend was reversed when the consultation would be with a familiar ophthalmologist—77.4% (n = 698) would proceed with it, while 15.1% (n = 136) expressed a negative attitude. Education was a significant factor (*p* = 0.003), with higher levels (Bachelor’s, Master’s, Doctorate) associated with greater willingness (see Table 3). A significant association was also found regarding age (*p* = 0.020), with younger individuals being more open to remote consultations (40.96 ± 12.264) (see Table 4). Regarding the type of settlement (*p* = 0.033), positive attitudes were similar (urban: 77.3%, rural: 78.6%), but respondents in rural areas showed higher levels of uncertainty.

In cases requiring long-term treatment of an eye condition, 55.2% (n = 498) expressed a positive attitude, while 31.1% (n = 280) responded negatively. A statistically significant association with gender was observed (*p* < 0.001): men (73.1%) were more positively inclined compared to women (51.5%) (see Table 2). Age (*p* < 0.001) and education level (*p* < 0.001) also had a significant impact—the proportion of positive responses increased with both age and educational attainment, particularly among respondents with a master’s degree (61.2%) and a doctorate (60.7%). Individuals with primary or secondary education were more skeptical and more likely to respond negatively (see Table 3 and Table 4).

**Figure 1:**
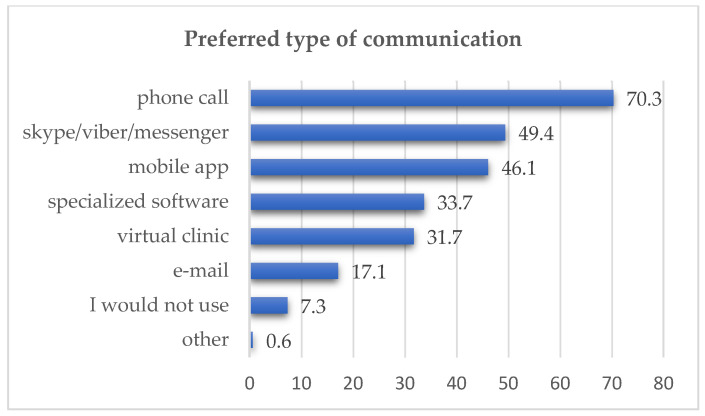
Preferred type of communication—users (in %).

In relation to the preferred mode of communication when using remote services, a total of 1674 responses were received from healthcare service users, as multiple answers were allowed. A significant portion selected ‘telephone call’ (n = 634; 70.3%); however, a considerable number also chose ‘mobile application’ (n = 416; 46.1%) and ‘specialized software’ (n = 304; 33.7%). Only 7.3% (n = 66) stated that they would not use any device. The data is presented in Figure 1.

**Figure 2:**
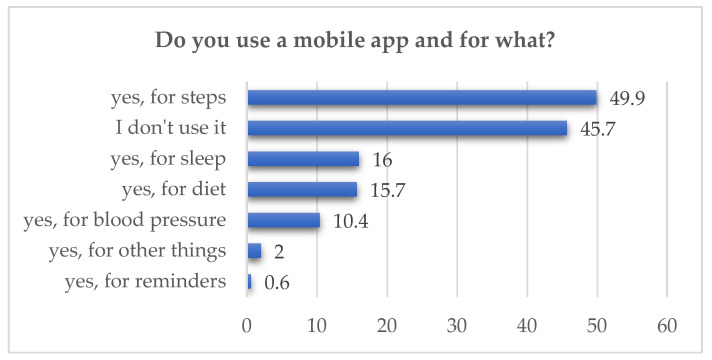
Mobile health applications reported by users (in %).

A significant proportion of respondents had already used mobile health applications. The data is presented in Figure 2.

Respondents were also asked whether they considered the use of mobile applications in the area of eye diseases to be beneficial. A total of 43.9% (n = 396) stated that they did not currently use such applications but supported the idea, while 16.5% (n = 148) disapproved, and 35.7% were undecided. Only 4% reported that they both used and approved of such applications. A statistically significant association was found with educational level (*p* = 0.002), with respondents holding a doctoral degree (85.7%) and a master’s degree (82.9%) showing the highest levels of support.

In response to the question about which eye condition they would use remote medical services for, respondents most frequently indicated the following: ‘for information only’ (68.2%), eye irritation (45.7%), red eyes (41.7%), and itching (40.1%). Additionally, 13.3% reported they would use the service for their children. Only 7.1% stated they would not use such services at all. Open-ended responses included the following: prescription, serious illness, and emergency.

**Figure 3:**
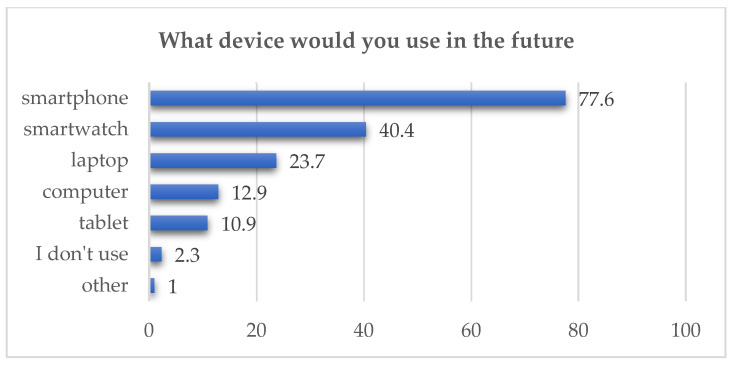
User preferences for future device use (in %).

The question on future use of digital devices for health monitoring revealed respondents’ digital literacy and attitudes toward mobile health apps. Smartphones were most frequently selected (77.6%). A total of 1524 responses were recorded (multiple choices allowed; see Figure 3).

In response to the question whether the implementation of specific digital solutions would improve communication between doctor and patient, a significant proportion (60.3%) of healthcare service users indicated that the addition of a real-time video call option would enhance such communication. A large number of responses also referred to telephone calls (51%) and a virtual office with video call functionality (46.3%), suggesting that personal contact (even virtual) was a key factor for respondents.

At the same time, in response to the question whether they believe treatment adherence would improve with enhanced doctor–patient communication, including via the use of digital technologies, 76.1% of surveyed healthcare service users answered positively, while 10.5% gave a negative response. This question was intentionally included, as it would be of critical importance and widely discussed in the context of glaucoma management.

Respondents were also asked whether treatment adherence would improve if awareness of the disease increased, including through the use of mobile applications. A significant proportion of healthcare service users responded positively, 80.1% (n = 722), while 7.5% (n = 68) gave negative responses. The most frequently cited source of information was the ophthalmologist (45.1%), followed by internet forums (23.6%), scientific publications (23.8%), friends/relatives (25.1%), and other medical professionals (14.1%). Health institution websites were used by 12.1% of respondents, and 9.3% cited education as their source of information.

In response to whether the use of artificial intelligence (AI) would improve doctor–patient interaction, 21.7% answered positively, 47.7% negatively, and 30.6% were undecided, indicating low awareness of the topic. A statistically significant association with gender was observed (*p* = 0.004): men were slightly more skeptical (48.8%) than women (47.7%), while women gave a slightly higher proportion of positive responses (27% compared to 20.6%).

Age also played a role (*p* < 0.001): the lowest average age was observed among those who responded negatively (37.34 years), while those with a positive attitude were, on average, 41 years old. A significant association was also found with educational level (*p* = 0.001): 100% of respondents with solely primary education rejected the usefulness of AI. Positive attitudes were most commonly observed among respondents with a doctoral degree (25%). The higher the level of education, the more positive the attitude toward AI. Regarding place of residence (*p* = 0.013), a higher proportion of uncertain responses was recorded among those living in Bulgaria (31.1%), suggesting lower awareness.

Regarding direct interaction with AI to learn more about their health status, 11.3% of participants reported having used AI, with 16 respondents indicating it was a regular practice. A total of 5.5% stated they were not familiar with technology, 81.4% had not used it, and 16.2% said they would not consider doing so.

**Figure 4:**
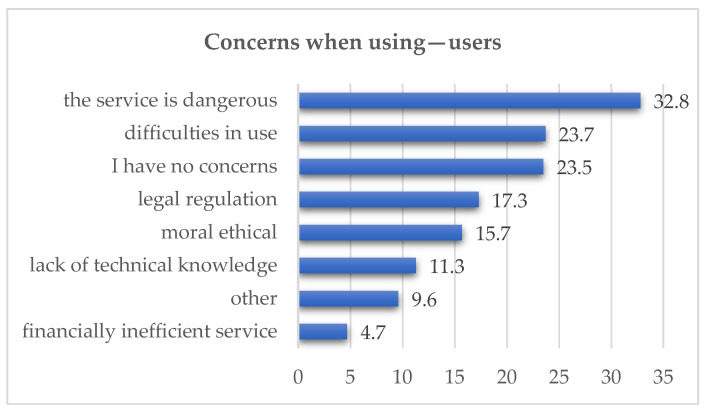
Concerns When Using—Users (in %).

Regarding concerns about using remote medical services, respondents were presented with multiple answer options. The largest number of participants indicated that such services carried risks (n = 296; 32.8%). A notable proportion also stated that they had no concerns (n = 212; 23.5%). A considerable number pointed to difficulties in using such services (n = 214; 23.7%). Concerns regarding legal regulation were expressed by 17.3% (n = 156), while 13.3% (n = 120) were worried about the misuse of personal data. In the ‘Other’ category, 96 respondents marked an answer, of whom 52 also provided a written opinion. The main concerns included inaccurate diagnosis and lack of appropriate equipment, cited most frequently, followed by lack of physical examination and personal contact. The data is presented in Figure 4.

One of the final questions in the survey addressed the overall opinion regarding the use of remote medical services.

**Figure 5:**
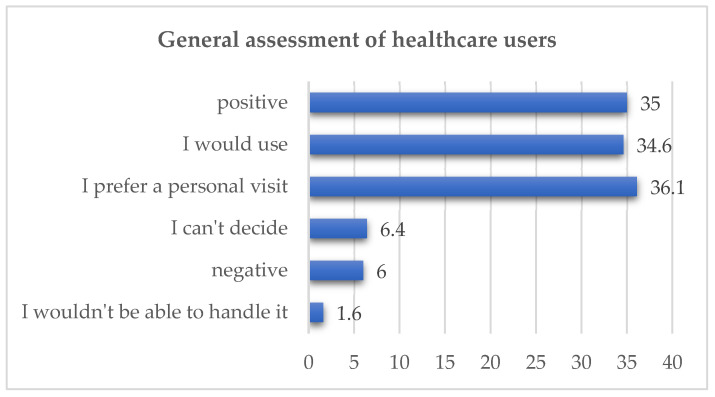
User opinion regarding telemedicine services (in %).

As multiple answers were allowed, a total of 1084 responses were collected from the group of healthcare service users. The number of positive responses was very high (n = 628; 69.6%), although some respondents expressed a negative attitude (n = 54; 6%). A substantial proportion also indicated a preference for in-person visits (n = 326; 36.1%). The data is presented in Figure 5.

The survey concluded with an open-ended question allowing respondents to share personal opinions or suggestions. Among healthcare service users, 74 responses were received, with most positive views expressed. Some highlighted that remote-access services could ensure timely contact with specialists in emergencies. Others valued technology as a complement to traditional exams, especially for consultations and preliminary symptom assessment. It was also noted that digital solutions were an inevitable part of the future but should be implemented alongside personal doctor-patient contact.

Alongside positive feedback, some respondents expressed reservations and concerns. These included the risk of missing important information in virtual exams that only physical check-ups can detect, difficulties elderly individuals may face using such services, and a preference for in-person visits.

## 4. Discussion

The results of the present study indicated a moderately positive attitude toward telemedicine, with 51.5% of respondents reporting willingness to use remote medical services. This proportion suggested that, despite evident interest, a substantial group of patients remained hesitant, likely reflecting the limited practical implementation of telemedicine services in Bulgaria at the time of the study. Higher acceptance was observed in more specific scenarios-62.5% would use telemedicine for consultation regarding an eye problem, and 55.2% for long-term treatment. These findings align with international studies, which reported that approximately 75% of patients are willing to use telemedicine, including teleglaucoma, with age and education influencing attitudes [7].

Similar results were reported in a 2019 study where it was found that patients with glaucoma demonstrated a high willingness to use telemedicine services, particularly when they already had established trust in their treating specialist [11]. Comparable observations were presented in 2022, according to which the attitude of glaucoma patients in Europe toward telemedicine was directly dependent on their digital literacy and previous experience with remote consultations [12]. The present study confirmed these trends, showing higher acceptance among younger and more educated participants, which may be explained by higher digital literacy and more frequent prior exposure to digital health technologies in these groups.

Similar positive trends had been observed in the United States, where, during the COVID-19 pandemic, over 7000 telephone consultations were recorded within a single month, most commonly related to acute eye symptoms and medication management [13]. A comparison with data from 2022 showed that telemedicine had already become the preferred option for 80% of users for the treatment of minor illnesses, prescription issuance, and initial consultations [14].

The preferred communication channel among respondents in our study was the telephone call (70.3%), followed by mobile applications (46.1%) and specialized software (33.7%). This is consistent with international data, where 57% of respondents preferred telephone communication, and 52% preferred video calls [15].

Despite the generally positive attitudes, there were notable concerns: 32.8% believed that telemedicine involved certain risks, and 23.7% reported difficulties in using it. These concerns highlighted the need for clearly defined protocols, patient education, and the assurance of quality in remote medical care. Similar concerns were reflected in international studies—for example, 42% of respondents in Rock Hills preferred in-person visits, and 22% feared a decline in the quality of care [16].

Mobile applications were viewed as a valuable tool—80.1% of respondents believed that they improved treatment adherence. This view was supported by international experience—for example, in China, 66.1% of respondents preferred mobile healthcare, with key factors being convenience, accessibility, and time-saving [17].

Educational level had a significant impact on attitudes—respondents with higher education demonstrated greater acceptance of telemedicine, mobile applications, and AI. Over 96% of participants with a doctoral degree knew what glaucoma was, compared to a significantly lower proportion among those with lower levels of education. This highlighted the need for targeted educational and informational campaigns aimed at improving health and digital literacy among vulnerable groups.

The results showed that 47.7% of respondents had a negative attitude toward AI, while 30.6% were undecided. Only 21.7% viewed AI as a useful tool in communication with a doctor. By comparison, in the United States, 55.4% of respondents believed that AI would improve healthcare, but 80% would prefer an in-person consultation for sensitive interventions [18,19].

Interestingly, 11.3% of our respondents had already used AI to obtain health information. This aligned with the growing trend of chatbot usage—according to a study involving 607 participants, 78.4% had used ChatGPT for self-diagnosis [20]. Another study with 64 patients found that ChatGPT even outperformed physicians in terms of empathy and usefulness in answering medical questions [21]. Although international studies reported widespread use of chatbots for self-diagnosis, the lower proportion observed in our results likely reflected lower digital health literacy and more limited access to such tools among Bulgarian patients.

AI capabilities go beyond diagnostics—it is able to assist in creating patient-tailored educational materials and support physicians in clinical decision-making [22,23].

Although attitudes toward telemedicine and digital health technologies were generally positive, important challenges remained. These included the need for greater awareness, technological support, regulatory frameworks, and trust. The successful integration of telemedicine and AI in ophthalmology depends on a balanced approach that combines technology, human presence, and the professional preparedness of all stakeholders involved in the process.

### Limitations of the Study

The present study was based on self-reported data which implies a potential degree of subjectivity in responses. The sample was not fully representative of the general population as the majority of respondents had higher education and resided in urban areas. The survey did not include a clinical assessment of visual status, and the glaucoma-related questions rely on self-reporting. Future research should aim to combine questionnaire data with objective clinical indicators and to expand the sample to include other socio-demographic groups.

Although the large sample size allows for more complex statistical modeling, the present study was not designed to test causal relationships. Future research should consider regression-based or structural modeling approaches to further explore the factors influencing teleophthalmology use.

## 5. Conclusions

The analysis of attitudes of health service users in Bulgaria showed that the perception of telemedicine and ophthalmology was predominantly positive, especially in the context of consultations for eye complaints and long-term follow-up of chronic diseases such as glaucoma. Despite the limited development and lack of systematic integration of teleophthalmological services in the country, a considerable part of the participants expressed willingness to use remote ophthalmological solutions in certain clinical situations.

The highest support was observed for remote consultations with familiar physicians and long-term eye condition monitoring. Willingness decreased in emergencies or when consulting unfamiliar specialists, underscoring the importance of trust in doctor–patient communication.

Socio-demographic factors, especially the educational level, influenced the attitudes towards telemedicine, mobile health applications, and the usage of artificial intelligence, which directed towards the necessity of purposeful informational and educational initiatives. Awareness of glaucoma was notably high among the highly educated, favoring teleophthalmology implementation for its monitoring.

Mobile applications and digital tools were perceived as useful for communication and treatment adherence. However, skepticism and limited awareness regarding AI highlighted the need to enhance trust and digital literacy among users.

In conclusion, evaluation of the patient attitudes presents an important first step towards the planning and future integration of teleophthalmological services in Bulgaria. The successful integration of telemedicine and digital technologies into ophthalmology requires a balanced approach that combines technological solutions and human presence, a clear regulatory framework, and adequate professional preparation.

## Figures and Tables

**Table 1:** Consumer attitudes towards the use of telemedicine services.

Questions		No	Somewhat No	Undecided	Somewhat Yes	Yes	Total
1. Would you use remote medical services/telemedicine?	n	44	192	202	256	208	902
	%	4.9	21.3	22.4	28.4	23.1	100.0
2. Would you use remote medical services to consult with a medical professional regarding a present eye issue?	n	64	156	118	286	278	902
	%	7.1	17.3	13.1	31.7	30.8	100.0
3. Would you use remote medical services for the prescription and commencement of treatment in relation to a present health-related eye issue?	n	100	224	142	246	190	902
	%	11.1	24.8	15.7	27.3	21.1	100.0
4. Do you agree to consult with an unfamiliar ophthalmologist via telephone, computer, or mobile application, instead of in person?	n	164	310	150	176	102	902
	%	18.2	34.4	16.6	19.5	11.3	100.0
5. Do you agree to consult with a familiar ophthalmologist using a telephone, computer, or mobile application, instead of in person?	n	52	84	68	310	388	902
	%	5.8	9.3	7.5	34.4	43.0	100.0
6. Do you agree to be monitored by a medical professional using a remote service during cont. treatment of an eye condition, instead of in person?	n	90	190	124	312	186	902
	%	10.0	21.1	13.7	34.6	20.6	100.0
7. Do you agree to be monitored by a medical professional via a remote service during an EMERGENCY eye condition, instead of in person?	n	252	262	110	172	106	902
	%	27.9	29.0	12.2	19.1	11.8	100.0

**Table 2:** Relationship between consumer attitudes towards the use of telemedicine services and gender.

Questions	Gender	Respondents’ Answers (n, %)					Total	*p*
		No	Somewhat No	Undecided	Somewhat Yes	Yes		
1	male	12 (7.7%)	20 (12.8%)	22 (14.1%)	56 (35.9%)	46 (29.5%)	156 (100.0%)	<0.001
	female	32 (4.3%)	172 (23.1%)	180 (24.1%)	200 (26.8%)	162 (21.7%)	746 (100.0%)	
	Total	44 (4.9%)	192 (21.3%)	202 (22.4%)	256 (28.4%)	208 (23.1%)	902 (100.0%)	
2	male	8 (5.1%)	16 (10.3%)	18 (11.5%)	52 (33.3%)	62 (39.7%)	156 (100.0%)	0.019
	female	56 (7.5%)	140 (18.8%)	100 (13.4%)	234 (31.4%)	216 (29.0%)	746 (100.0%)	
	Total	64 (7.1%)	156 (17.3%)	118 (13.1%)	286 (31.7%)	278 (30.8%)	902 (100.0%)	
3	male	10 (6.4%)	34 (21.8%)	24 (15.4%)	46 (29.5%)	42 (26.9%)	156 (100.0%)	0.097
	female	90 (12.1%)	190 (25.5%)	118 (15.8%)	200 (26.8%)	148 (19.8%)	746 (100.0%)	
	Total	100 (11.1%)	224 (24.8%)	142 (15.7%)	246 (27.3%)	190 (21.1%)	902 (100.0%)	
4	male	28 (17.9%)	36 (23.1%)	20 (12.8%)	48 (30.8%)	24 (15.4%)	156 (100.0%)	<0.001
	female	136 (18.2%)	274 (36.7%)	130 (17.4%)	128 (17.2%)	78 (10.5%)	746 (100.0%)	
	Total	164 (18.2%)	310 (34.4%)	150 (16.6%)	176 (19.5%)	102 (11.3%)	902 (100.0%)	
5	male	8 (5.1%)	10 (6.4%)	12 (7.7%)	44 (28.2%)	82 (52.6%)	156 (100.0%)	0.094
	female	44 (5.9%)	74 (9.9%)	56 (7.5%)	266 (35.7%)	306 (41.0%)	746 (100.0%)	
	Total	52 (5.8%)	84 (9.3%)	68 (7.5%)	310 (34.4%)	388 (43.0%)	902 (100.0%)	
6	male	10 (6.4%)	18 (11.5%)	14 (9.0%)	66 (42.3%)	48 (30.8%)	156 (100.0%)	<0.001
	female	80 (10.7%)	172 (23.1%)	110 (14.7%)	246 (33.0%)	138 (18.5%)	746 (100.0%)	
	Total	90 (10.0%)	190 (21.1%)	124 (13.7%)	312 (34.6%)	186 (20.6%)	902 (100.0%)	
7	male	32 (20.5%)	38 (24.4%)	20 (12.8%)	38 (24.4%)	28 (17.9%)	156 (100.0%)	0.007
	female	220 (29.5%)	224 (30.0%)	90 (12.1%)	134 (18.0%)	78 (10.5%)	746 (100.0%)	
	Total	252 (27.9%)	262 (29.0%)	110 (12.2%)	172 (19.1%)	106 (11.8%)	902 (100.0%)	

**Table 3:** Relationship between consumer attitudes towards the use of telemedicine services and level of education.

Questions	Level of Education	Respondents’ Answers (n, %)					Total	*p*
		No	Somewhat No	Undecided	Somewhat Yes	Yes		
1	primary	2 (50.0%)	0 (0.0%)	2 (50.0%)	0 (0.0%)	0 (0.0%)	4 (100.0%)	<0.001
	secondary	16 (7.1%)	44 (19.6%)	48 (21.4%)	56 (25.0%)	60 (26.8%)	224 (100.0%)	
	bachelor	12 (5.2%)	50 (21.6%)	62 (26.7%)	62 (26.7%)	46 (19.8%)	232 (100.0%)	
	master	14 (3.6%)	82 (21.2%)	86 (22.3%)	124 (32.1%)	80 (20.7%)	386 (100.0%)	
	doctoral degree	0 (0.0%)	16 (28.6%)	4 (7.1%)	14 (25.0%)	22 (39.3%)	56 (100.0%)	
	Total	44 (4.9%)	192 (21.3%)	202 (22.4%)	256 (28.4%)	208 (23.1%)	902 (100.0%)	
2	primary	2 (50.0%)	0 (0.0%)	0 (0.0%)	0 (0.0%)	2 (50.0%)	4 (100.0%)	0.006
	secondary	20 (8.9%)	44 (19.6%)	32 (14.3%)	58 (25.9%)	70 (31.3%)	224 (100.0%)	
	bachelor	16 (6.9%)	48 (20.7%)	32 (13.8%)	76 (32.8%)	60 (25.9%)	232 (100.0%)	
	master	26 (6.7%)	52 (13.5%)	44 (11.4%)	136 (35.2%)	128 (33.2%)	386 (100.0%)	
	doctoral degree	0 (0.0%)	12 (21.4%)	10 (17.9%)	16 (28.6%)	18 (32.1%)	56 (100.0%)	
	Total	64 (7.1%)	156 (17.3%)	118 (13.1%)	286 (31.7%)	278 (30.8%)	902 (100.0%)	
3	primary	2 (50.0%)	0 (0.0%)	2 (50.0%)	0 (0.0%)	0 (0.0%)	4 (100.0%)	0.001
	secondary	32 (14.3%)	70 (31.3%)	32 (14.3%)	44 (19.6%)	46 (20.5%)	224 (100.0%)	
	bachelor	26 (11.2%)	60 (25.9%)	34 (14.7%)	56 (24.1%)	56 (24.1%)	232 (100.0%)	
	master	32 (8.3%)	82 (21.2%)	64 (16.6%)	134 (34.7%)	74 (19.2%)	386 (100.0%)	
	doctoral degree	8 (14.3%)	12 (21.4%)	10 (17.9%)	12 (21.4%)	14 (25.0%)	56 (100.0%)	
	Total	100 (11.1%)	224 (24.8%)	142 (15.7%)	246 (27.3%)	190 (21.1%)	902 (100.0%)	
4	primary	2 (50.0%)	2 (50.0%)	0 (0.0%)	0 (0.0%)	0 (0.0%)	4 (100.0%)	0.294
	secondary	50 (22.3%)	72 (32.1%)	36 (16.1%)	38 (17.0%)	28 (12.5%)	224 (100.0%)	
	bachelor	40 (17.2%)	74 (31.9%)	32 (13.8%)	54 (23.3%)	32 (13.8%)	232 (100.0%)	
	master	60 (15.5%)	142 (36.8%)	74 (19.2%)	72 (18.7%)	38 (9.8%)	386 (100.0%)	
	doctoral degree	12 (21.4%)	20 (35.7%)	8 (14.3%)	12 (21.4%)	4 (7.1%)	56 (100.0%)	
	Total	164 (18.2%)	310 (34.4%)	150 (16.6%)	176 (19.5%)	102 (11.3%)	902 (100.0%)	
5	primary	2 (50.0%)	0 (0.0%)	0 (0.0%)	0 (0.0%)	2 (50.0%)	4 (100.0%)	0.003
	secondary	20 (8.9%)	20 (8.9%)	24 (10.7%)	72 (32.1%)	88 (39.3%)	224 (100.0%)	
	bachelor	14 (6.0%)	24 (10.3%)	16 (6.9%)	74 (31.9%)	104 (44.8%)	232 (100.0%)	
	master	16 (4.1%)	38 (9.8%)	22 (5.7%)	140 (36.3%)	170 (44.0%)	386 (100.0%)	
	doctoral degree	0 (0.0%)	2 (3.6%)	6 (10.7%)	24 (42.9%)	24 (42.9%)	56 (100.0%)	
	Total	52 (5.8%)	84 (9.3%)	68 (7.5%)	310 (34.4%)	388 (43.0%)	902 (100.0%)	
6	primary	2 (50.0%)	0 (0.0%)	2 (50.0%)	0 (0.0%)	0 (0.0%)	4 (100.0%)	<0.001
	secondary	24 (10.7%)	60 (26.8%)	34 (15.2%)	60 (26.8%)	46 (20.5%)	224 (100.0%)	
	bachelor	32 (13.8%)	46 (19.8%)	32 (13.8%)	74 (31.9%)	48 (20.7%)	232 (100.0%)	
	master	32 (8.3%)	72 (18.7%)	46 (11.9%)	160 (41.5%)	76 (19.7%)	386 (100.0%)	
	doctoral degree	0 (0.0%)	12 (21.4%)	10 (17.9%)	18 (32.1%)	16 (28.6%)	56 (100.0%)	
	Total	90 (10.0%)	190 (21.1%)	124 (13.7%)	312 (34.6%)	186 (20.6%)	902 (100.0%)	
7	primary	2 (50.0%)	0 (0.0%)	0 (0.0%)	0 (0.0%)	2 (50.0%)	4 (100.0%)	0.029
	secondary	78 (34.8%)	66 (29.5%)	22 (9.8%)	32 (14.3%)	26 (11.6%)	224 (100.0%)	
	bachelor	66 (28.4%)	72 (31.0%)	28 (12.1%)	40 (17.2%)	26 (11.2%)	232 (100.0%)	
	master	94 (24.4%)	114 (29.5%)	50 (13.0%)	86 (22.3%)	42 (10.9%)	386 (100.0%)	
	doctoral degree	12 (21.4%)	10 (17.9%)	10 (17.9%)	14 (25.0%)	10 (17.9%)	56 (100.0%)	
	Total	252 (27.9%)	262 (29.0%)	110 (12.2%)	172 (19.1%)	106 (11.8%)	902 (100.0%)	

**Table 4:** Relationship between consumer attitudes towards the use of telemedicine services and age.

Questions Number		Mean (Age)	N	SD	*p*
1	no	43.45	44	18.182	0.014
	somewhat no	39.06	192	13.068	
	undecided	39.09	202	11.480	
	somewhat yes	42.00	256	11.241	
	yes	41.54	208	12.927	
	Total	40.69	902	12.558	
2	no	43.50	64	15.954	0.001
	somewhat no	37.37	156	11.736	
	undecided	38.98	118	11.016	
	somewhat yes	41.43	286	11.571	
	yes	41.86	278	13.332	
	Total	40.69	902	12.558	
3	no	40.40	100	14.022	<0.001
	somewhat no	37.90	224	12.386	
	undecided	40.83	142	11.805	
	somewhat yes	43.37	246	11.312	
	yes	40.54	190	13.389	
	Total	40.69	902	12.558	
4	no	40.29	164	13.865	0.588
	somewhat no	40.05	310	12.576	
	undecided	40.64	150	11.947	
	somewhat yes	41.39	176	11.862	
	yes	42.12	102	12.407	
	Total	40.69	902	12.558	
5	no	44.46	52	13.775	0.020
	somewhat no	37.74	84	12.750	
	undecided	38.59	68	13.510	
	somewhat yes	40.90	310	12.179	
	yes	41.02	388	12.349	
	Total	40.69	902	12.558	
6	no	39.98	90	13.872	<0.001
	somewhat no	37.14	190	12.427	
	undecided	38.89	124	12.211	
	somewhat yes	42.33	312	11.802	
	yes	43.10	186	12.593	
	Total	40.69	902	12.558	
7	no	37.98	252	12.306	<0.001
	somewhat no	40.14	262	11.559	
	undecided	42.76	110	12.627	
	somewhat yes	42.88	172	13.418	
	yes	42.77	106	12.849	
	Total	40.69	902	12.558	

## Data Availability

The original contributions presented in this study are included in the article/Supplementary Materials. Further inquiries can be directed to the corresponding author(s).

## Supplementary Materials

The following supporting information can be downloaded at: https://www.mdpi.com/article/10.3390/healthcare14020273/s1, File S1: Questionnaire on Attitudes Toward Teleophthalmology.
