# Supplementary material for: Attitudes of Healthcare Service Users in Bulgaria Towards the Application of Teleophthalmology in the Case of Glaucoma
Source: Healthcare (Basel). 2026 Jan 21;14(2):273. doi: 10.3390/healthcare14020273 (PMC12840610; doi:10.3390/healthcare14020273)
Supplement: Supplementary file 1 [file healthcare-14-00273-s001.zip › healthcare-4056661-supplementary.pdf]

Supplementary File S1.
Questionnaire on Attitudes Toward Teleophthalmology

The purpose of this survey is to explore public attitudes toward the use of remote medical services in the context of eye diseases.
The survey is anonymous and does not collect personal data. The results will be used solely for research purposes.
Please mark your answer by placing an “X” in the appropriate circle. Only one answer is allowed for each question unless otherwise specified. Please provide written responses where indicated.
Thank you for your time and participation!

Sociodemographic Information

Gender:
☐ Male ☐ Female

Age (years): __________

Education level:
☐ Primary
☐ Secondary
☐ Bachelor’s degree
☐ Master’s degree
☐ Doctoral degree

Place of residence:
☐ Urban ☐ Rural

Do you live and work in Bulgaria?
☐ Yes ☐ No

Have you been diagnosed with an eye condition?
☐ Yes ☐ No

Questionnaire Items

1. Would you use remote medical services (telemedicine)?

☐ Yes
☐ Rather yes
☐ Cannot assess
☐ Rather no
☐ No

2. Have you ever used remote medical services?

☐ Yes
☐ No, but I plan to use them in the future
☐ I am not familiar with such services
☐ No

If "Yes", please answer Questions 2.1–2.5.

2.1 For what purpose did you use remote medical services?

(Multiple answers allowed)

☐ Consultation
☐ Information only
☐ Second opinion
☐ Lifestyle recommendations
☐ Treatment
☐ Other (please specify): ________________________

2.2 How was the telemedical consultation conducted?

(Multiple answers allowed)

☐ Telephone
☐ Skype
☐ Viber
☐ Email
☐ Specialized software
☐ Virtual clinic
☐ Mobile application
☐ Other: ________________________

2.3 Which specialist did you consult? Please specify the specialty:

2.4 Were you satisfied with the telemedical consultation?

☐ Yes
☐ Rather yes
☐ Cannot assess
☐ Rather no
☐ No

2.5 Did you know the physician you consulted remotely?

☐ Yes
☐ Rather yes
☐ Cannot assess
☐ Rather no
☐ No

3. Would you use remote medical services to consult a healthcare professional (physician, nurse, etc.) regarding an eye-related health problem?

☐ Yes
☐ Rather yes
☐ Cannot assess
☐ Rather no
☐ No

4. Would you use remote medical services for prescribing and initiating treatment for an eye-related health problem?

☐ Yes
☐ Rather yes
☐ Cannot assess
☐ Rather no
☐ No

5. Would you agree to consult an ophthalmologist you do not know via phone, computer, or mobile application instead of an in-person visit?

☐ Yes
☐ Rather yes
☐ Cannot assess
☐ Rather no
☐ No

6. Would you agree to consult an ophthalmologist you know via phone, computer, or mobile application instead of an in-person visit?

☐ Yes
☐ Rather yes
☐ Cannot assess
☐ Rather no
☐ No

7. Would you use information from the internet to determine what condition you may have instead of visiting a physician?

☐ Yes
☐ Rather yes
☐ Cannot assess
☐ Rather no
☐ No

8. Would you search for information on the internet to learn more about a diagnosis already made by an ophthalmologist?

☐ Yes
☐ Rather yes
☐ Cannot assess
☐ Rather no
☐ No

9. Would you agree to be monitored remotely by a healthcare professional in the case of long-term treatment for an eye disease instead of in-person visits?

☐ Yes
☐ Rather yes
☐ Cannot assess
☐ Rather no
☐ No

10. Would you agree to be consulted remotely by a healthcare professional in the case of an ophthalmic emergency instead of an in-person visit?

☐ Yes
☐ Rather yes
☐ Cannot assess
☐ Rather no
☐ No

11. Do you think remote medical services should be less expensive than standard in-person visits?

☐ Yes
☐ Rather yes
☐ Cannot assess
☐ Rather no
☐ No

12. Do you think remote medical services should be covered by the national health insurance fund rather than paid directly by the patient?

☐ Yes
☐ Rather yes
☐ Cannot assess
☐ Rather no
☐ No

13. Do you use health-related applications on your phone or tablet?

(Multiple answers allowed)

☐ Yes – blood pressure monitoring
☐ Yes – sleep tracking
☐ Yes – step counting
☐ Yes – eye-related applications (e.g., visual acuity charts)
☐ Yes – dietary tracking
☐ Other: ______________________
☐ No

14. Which device do you use or would you use in the future to monitor your health status?

(Multiple answers allowed)

☐ Desktop computer
☐ Laptop
☐ Tablet
☐ Smartphone
☐ Smartwatch
☐ Other: ______________________

15. On average, how many hours per day do you use digital devices (computer, laptop, tablet, smartphone, smartwatch)?

☐ Less than 2 hours
☐ 2–4 hours
☐ 4–8 hours
☐ More than 8 hours
☐ I do not use them every day

16. For which eye-related conditions would you use remote medical services if available?

(Multiple answers allowed)

☐ Information only
☐ Eye irritation
☐ Itching
☐ Blurred vision
☐ Foreign body sensation
☐ Red eyes
☐ For my child
☐ Other: ______________________

17. Which of the following would you use to consult a healthcare professional remotely?

(Multiple answers allowed)

☐ Telephone call
☐ Skype
☐ Messenger
☐ Specialized software
☐ Mobile application
☐ Email
☐ Virtual clinic
☐ Other: ______________________

18. What concerns do you have regarding the use of remote medical services?

(Multiple answers allowed)

☐ Ethical concerns
☐ Legal regulation
☐ Lack of technical skills
☐ Potential risks of the service
☐ Financial inefficiency
☐ No concerns
☐ Misuse of personal data
☐ Difficulties in use
☐ Other: ______________________

19. What concerns do you have when searching for health-related information on the internet?

(Multiple answers allowed)

☐ Risk of incorrect treatment
☐ Unclear source of information
☐ Technical difficulties
☐ Incorrectly presented information
☐ I do not read such information
☐ I am unsure whether the information applies to me
☐ No concerns
☐ Other: ______________________

20. Do you know what glaucoma is?

☐ Yes
☐ Rather yes
☐ Cannot assess
☐ Rather no
☐ No

If "Yes", what is your source of information?
(Multiple answers allowed)

☐ Ophthalmologist
☐ Other healthcare professional
☐ Healthcare institution website
☐ Internet website, forum, or blog
☐ Family member or friend
☐ Social media
☐ Scientific publications
☐ Radio or television
☐ Printed media
☐ Other: ______________________

21. Do you have any of the following?

(Multiple answers allowed)

☐ Blood relative with glaucoma
☐ Myopia
☐ Glaucoma
☐ Previously measured elevated intraocular pressure
☐ Low blood pressure
☐ Eye trauma
☐ Long-term corticosteroid use
☐ None

22. When was your last visit to an ophthalmologist?

☐ Within the last 3 months
☐ 3–12 months ago
☐ Up to 3 years ago
☐ Up to 5 years ago
☐ I have not visited since childhood
☐ I have never visited

23. Which of the following would you find useful in communication with a physician or healthcare professional?

(Multiple answers allowed)

☐ Real-time video consultation
☐ Telephone call
☐ Email correspondence
☐ Text messages (SMS)
☐ Images (e.g., photos) sent electronically
☐ Virtual clinic with video consultation option
☐ Sharing online materials related to your condition
☐ Other: ______________________
☐ None

24. Do you find mobile applications in the field of ophthalmology useful for patients?

☐ Yes, I use such applications
☐ Yes, I do not use them, but I support the idea
☐ Cannot assess
☐ Rather no
☐ No

25. Do you think your adherence to prescribed treatment would improve if communication opportunities between physician and patient were expanded, including through digital technologies?

☐ Yes
☐ Rather yes
☐ Cannot assess
☐ Rather no
☐ No

26. Do you think your adherence to prescribed treatment would improve if your awareness of the disease increased, including through the use of mobile applications?

☐ Yes
☐ Rather yes
☐ Cannot assess
☐ Rather no
☐ No

27. Do you think artificial intelligence (e.g., ChatGPT) can support doctor–patient interaction?

☐ Yes
☐ Rather yes
☐ Cannot assess
☐ Rather no
☐ No

28. Have you ever interacted with artificial intelligence (e.g., ChatGPT) to ask questions about your health condition?

☐ Yes, regularly
☐ Yes
☐ I am not familiar with it
☐ No
☐ I would not do so

29. What is your overall attitude toward using medical services via a digital device for information and/or remote consultation?

(Multiple answers allowed)

☐ Positive
☐ I would use such services
☐ I would not be able to manage
☐ I prefer in-person visits
☐ Negative
☐ Cannot assess
☐ Other: ______________________

Additional comments or recommendations regarding this survey:

